# Quantitative proteomics reveals cellular responses to individual mAb expression and tunicamycin in CHO cells

**DOI:** 10.1007/s00253-024-13223-1

**Published:** 2024-06-19

**Authors:** Eldi Sulaj, Linda Schwaigerlehner, Felix L. Sandell, Juliane C. Dohm, Gorji Marzban, Renate Kunert

**Affiliations:** 1 Department of Biotechnology, Institute of Animal Cell Technology and Systems Biology (IACTSB), BOKU University, Muthgasse 18, 1190 Vienna, Austria; 2 Department of Biotechnology, Institute of Computational Biology (ICB), BOKU University, Muthgasse 18, 1190 Vienna, Austria; 3 Department of Biotechnology, Institute of Bioprocess Science and Engineering (IBSE), BOKU University, Muthgasse 18, 1190 Vienna, Austria

**Keywords:** Chinese hamster ovary, Proteome, LFQ, Tunicamycin, ER stress

## Abstract

**Abstract:**

Chinese hamster ovary (CHO) cells are popular in the pharmaceutical industry for their ability to produce high concentrations of antibodies and their resemblance to human cells in terms of protein glycosylation patterns. Current data indicate the relevance of CHO cells in the biopharmaceutical industry, with a high number of product commendations and a significant market share for monoclonal antibodies. To enhance the production capabilities of CHO cells, a deep understanding of their cellular and molecular composition is crucial. Genome sequencing and proteomic analysis have provided valuable insights into the impact of the bioprocessing conditions, productivity, and product quality. In our investigation, we conducted a comparative analysis of proteomic profiles in high and low monoclonal antibody–producing cell lines and studied the impact of tunicamycin (TM)-induced endoplasmic reticulum (ER) stress. We examined the expression levels of different proteins including unfolded protein response (UPR) target genes by using label-free quantification techniques for protein abundance. Our results show the upregulation of proteins associated with protein folding mechanisms in low producer vs. high producer cell line suggesting a form of ER stress related to specific protein production. Further, Hspa9 and Dnaja3 are notable candidates activated by the mitochondria UPR and play important roles in protein folding processes in mitochondria. We identified significant upregulation of Nedd8 and Lgmn proteins in similar levels which may contribute to UPR stress. Interestingly, the downregulation of Hspa5/Bip and Pdia4 in response to tunicamycin treatment suggests a low-level UPR activation.

**Key points:**

*• Proteome profiling of recombinant CHO cells under mild TM treatment.*

*• Identified protein clusters are associated with the unfolded protein response (UPR).*

*• The compared cell lines revealed noticeable disparities in protein expression levels.*

**Supplementary Information:**

The online version contains supplementary material available at 10.1007/s00253-024-13223-1.

## Introduction

The study of Chinese hamster ovary (CHO) cells has been conducted extensively since their isolation in the mid 1950s (Puck 1957). Over the years, CHO cells have remained a popular choice in the pharmaceutical industry due to their ability to produce high concentrations of antibodies, reaching up to 10 g/L (Reinhart et al. 2015). Moreover, they demonstrate the closest resemblance to human cells in terms of the reconstruction of protein glycosylation patterns (Hossler et al. 2009; Sasaki et al. 1988; Yang et al. 2015).

Regularly updated data reveal the ongoing relevance of CHO cells in the pharmaceutical industry. Between January 2018 and June 2022, a total of 189 product commendations were made in the biopharmaceutical industry (Walsh and Walsh 2022). Notably, 85 of these commendations were for novel entries, indicating the ongoing interest and development in the field (Walsh and Walsh 2022). Among these commendations, monoclonal antibodies accounted for more than 50%, with a market share valued at 80% or $217 billion in total protein sales for the previous year (Walsh and Walsh 2022). These figures surpassed even the most optimistic financial sales projections made before, underlining the significance of the biopharmaceutical market and the pivotal role played by CHO cells (Ecker et al. 2015). To enhance the production capabilities and efficiency of CHO cells, a profound understanding of their cellular and molecular composition is crucial. This understanding is essential for leveraging their potential and further advancing their role in the biopharmaceutical industry.

Since the initial genome sequencing of CHO cells (Xu et al. 2011), subsequent efforts focused on refining the genome sequences (Kaas et al. 2015; Lewis et al. 2013). These endeavors laid the foundation for proteomic analysis, uncovering numerous insights about the impact of entire proteome and specific bioprocessing conditions on productivity and product quality (Heffner et al. 2017; Sommeregger et al. 2016; Strasser et al. 2021; Xu et al. 2017). Additionally, several studies have examined the impact of cell culture conditions such as hyperosmolality, media composition, and cell physiological state on the protein expression (Kaushik et al. 2020; Romanova et al. 2022; Yu et al. 2022). Despite this, advancements and refinements in technological developments for instrumental analytics, (Williamson et al. 2016), genomic databases (Rupp et al. 2018), and tools for proteomic analysis (Bloom et al. 2021; Didusch et al. 2022; Gallant et al. 2020; Shah et al. 2020; Tyanova and Cox 2018) continue to progress each year, leaving ample space for further improvements.

At the forefront of ongoing and past research efforts, scientists are dedicated to achieving a stable mammalian cell line that can consistently produce authentic antibodies. This accomplishment is considered the epitome of success in this field. However, until this milestone is reached, any progress made must carefully consider the delicate balance between improving production quality, quantity, and cost-effectiveness (Barnes et al. 2003; Dorai et al. 2012). The field of proteomics has now reached a stage where it can identify and quantify thousands of cellular proteins, facilitating a deeper understanding of cell physiology (Heffner et al. 2017).

To elucidate differences between good and bad producers at the proteomics level, we proposed a plausible hypothesis for low production, suggesting that the low producing antibody generates cellular stress (due to formation of aggregates or dimers), consequently leading to downregulation of monoclonal antibody (mAb) expression, as observed in the case of 2G12 (Schwaigerlehner et al. 2019). As shown in the paper (Schwaigerlehner et al. 2019), we compared the affinity-matured naturally occurring antibody 2G12 (low producer) with its corresponding germline variant 353/11 (high producer). The data show significant differences in the CDR-H3 region, which is three amino acids longer than the closest germline sequence and only matches in 4 out of 14 amino acids. This is attributed to the fact that CDR-H3 regions are determined by germline D-regions which undergo inaccuracies in V-D and D-J rearrangement to increase divergence. Furthermore, V_H_ regions of the 2G12 and 353/11 sequences revealed more deviations than V_L_, differing in hydropathy and solubility (aggregation potential). Hydropathy index differed in distinct regions of the variable regions between the matured antibody and the germline sequences of the two antibodies, and spatial aggregation propensity (SAP) indicated more aggregation-prone patches in 2G12 compared to 353/11. Moreover, individual amino acids occurred in varying numbers in the variable region of 2G12 and 353/11. Histidine, proline, and phenylalanine are increased in 2G12, and tyrosine, serine, and tryptophan in 353/11 mAb, which are suspected to have an influence on the ease of production.

In our investigation, we conducted a comparative analysis of the proteomic profiles of two cell lines expressing different mAbs at varying production levels (low and high producing cell lines). Additionally, we investigated the impact of tunicamycin (TM), a compound known for its ability to block protein glycosylation by inhibiting the initial step in the lipid-linked pathway (Elbein 1987), which induces endoplasmic reticulum (ER) stress. Our investigation attempts to understand the effects of TM-induced ER stress on the cellular response, particularly focusing on the unfolded protein response (UPR). To evaluate this, we examined the expression levels of some well-known UPR target genes that are triggered by ER stress (Guillemette et al. 2007; Travers et al. 2000). For quantification of protein abundance, we employed label-free quantification (LFQ) techniques, enabling us to obtain comprehensive and unbiased measurements.

## Materials and methods

### Cell lines and cultivation regimes

The establishment of genetically identical CHO cell lines producing IgG antibodies was described before (Schwaigerlehner et al. 2019). In this study, two genetically modified CHO-K1 cell line cultivars expressing two specific human antibodies, namely 2G12 and 353/11, were used. The cells were cultured in chemically defined CD-CHO medium (Gibco, no. 10743–029, Grand Island, NY, USA), supplemented with 4 mM L-glutamine (Roth, no. 9183.1, Karlsruhe, Germany), 15 mg/L phenol red (Sigma-Aldrich, no. P0290, Schnelldorf, Germany), and 2 μM ganciclovir (GCV) (Sigma-Aldrich, no. G2536-100MG, Schnelldorf, Germany). To maintain consistent growth conditions and ensure a similar physiological state, a semi-perfusion process was employed using 50 ml vent cap spin tubes (Corning, no. 431720, Corning, NY, USA) placed in an ISF-X shaker (Kühner, Basel, Switzerland). The cells were incubated at 37 °C, 80% humidity, 5% CO_2_, and 220 rpm. The initial cell seeding density was 5 × 10^6^ cells per ml. Daily monitoring of cell counts and viability were conducted with a Vi-Cell XR Cell Counter (Beckman Coulter, Brea, CA, USA). Regular medium exchanges were carried out daily. This entailed the daily renewal of the media to uphold optimal cell viabilities and ensure procedural consistency. This approach was adopted to prevent any potential bias during the comparative study.

**Fig. 1:**
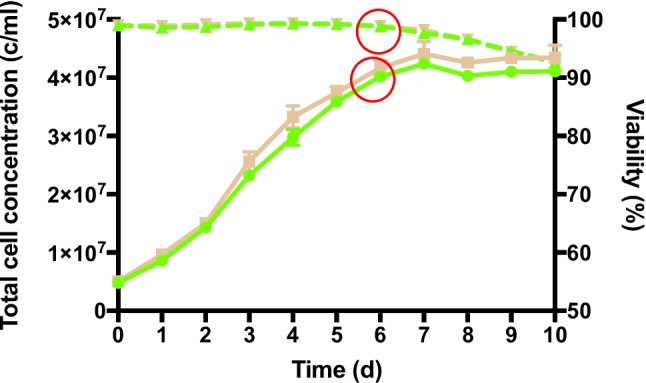
Cell growth across 353/11 cell line (shown in green) and 2G12 cell line (shown in brown), samples monitored by total cell concentration (straight line), and viability (dotted lines). Day 6 values are circled and pointed in red. (The graph was generated using GraphPad Prism 8.0, Boston, MA, USA, www.graphpad.com.)

To conduct proteomics analysis aimed at evaluating the differences between CHO cell lines expressing 2G12 and 353/11, cells were cultured using a semi-perfusion batch process in parallel. The cells were collected and pelleted on day 6, 4 h after medium exchange, when both cell lines had reached maximum density and exhibited sustained viability (Fig. 1). The treatment time was 4 h since our experimental approach was to take samples of all cultivars at the same time point after medium exchange. Sampling time point was defined as 4 h after the daily medium exchange either to bring them into optimal physiological conditions or to treat them with TM. One cell line exhibited high production (353/11), whereas the other one (2G12) displayed low production. To induce endoplasmic reticulum (ER) stress, 353/11 cells were cultivated separately and parallelly treated with tunicamycin (TM) at a final concentration of 1 µg/ml in fresh cultivation medium for 4 h prior to harvesting. This approach aimed to establish uniform experimental conditions comparable to those of non-treated cells and to identify early indicators or precursors of cellular stress. The harvested cells were centrifuged at 1300 rpm for 7 min at room temperature (RT); the pellets were washed twice in PBS buffer and subsequently stored at − 80 °C.

### Sample preparation

**Fig. 2:**
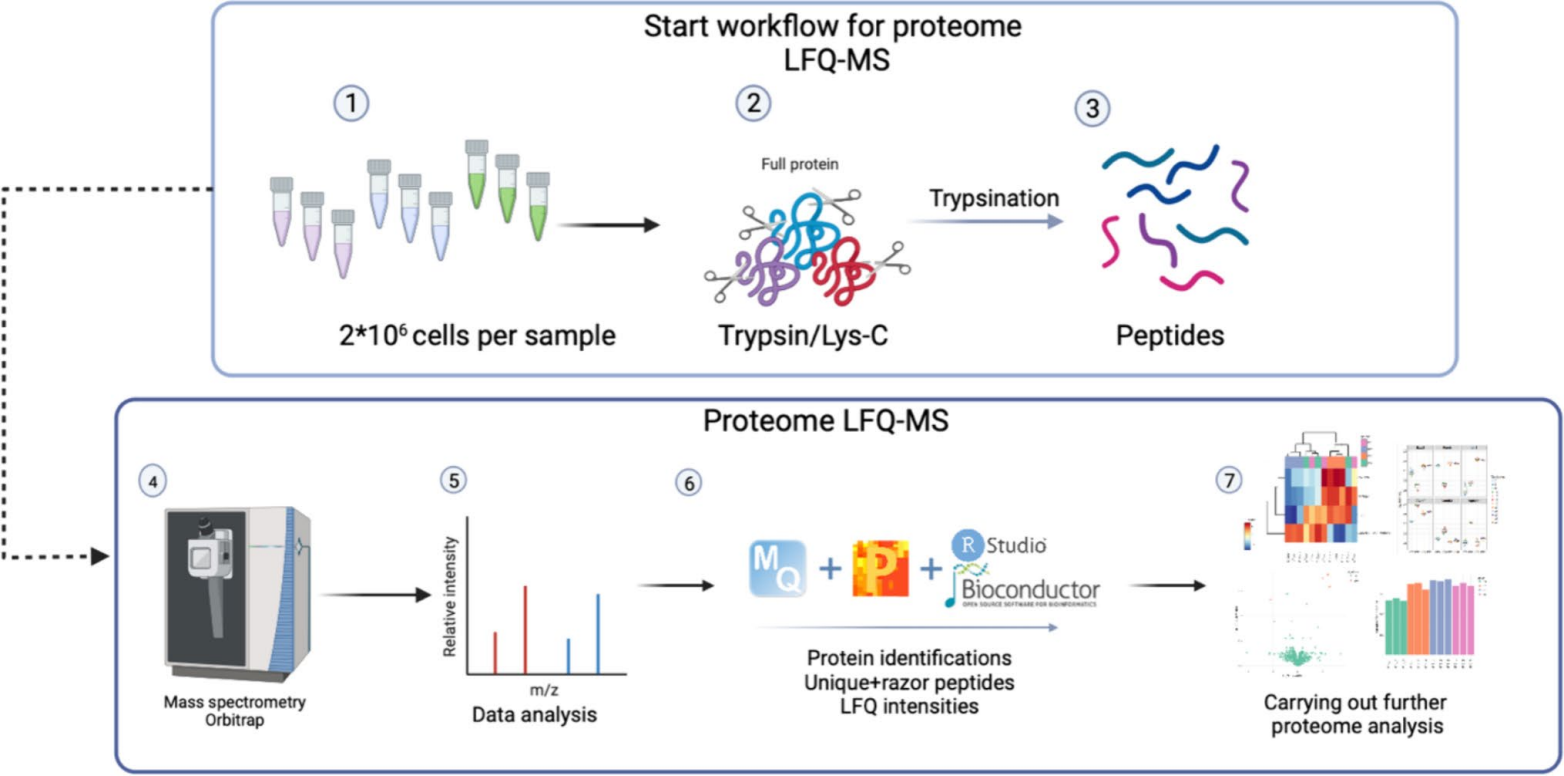
Schematic workflow of label-free quantification mass spectrometry (LFQ-MS) used for Chinese hamster ovary cells (CHO-K1) samples. (Created using BioRender.com.) The procedure comprises the following sequential steps: protein extraction, lysis, reduction, alkylation, enzymatic digestion, LC–MS/MS analysis, protein identification, and computational in silico analysis

For total proteome analysis, 2 × 10^6^ of the pelleted frozen cells were used (Fig. 1). The complete workflow, providing an overview of proteome analysis and the sample preparation process, is illustrated in Fig. 2. The schematic representation of this workflow was created using BioRender.com.

Two biological replicates were used for each of the three cell line groups. Each biological replicate consisted of three technical replicates, resulting in a total of 18 replicates. Each cell pellet composed of 2 × 10^6^ cells was resuspended in a 200-µl mixture of 50 mM tetraethylammonium bromide (TEAB) (Sigma-Aldrich, no. T7408, Schnelldorf, Germany) and 8 M urea buffer (pH 8.0) (Sigma-Aldrich, no. 51456, Schnelldorf, Germany) and transferred into a new low protein binding microcentrifuge tube (Thermo Fisher Scientific, no. 88379, Rockford, IL, USA). Then, 2 µl of protease and phosphatase inhibitor single-use cocktail (EDTA-free solution) (Thermo Fisher Scientific, no. 78443, Rockford, IL, USA) was added. The samples were lysed in an ice-cold Branson 2510 ultrasonic bath (Emerson Electric, Saint Louis, MO, USA) for 30 min at the standard frequency of 40 kHz. After sonification, the samples were diluted with an additional 200 µl of 50 mM TEAB and centrifuged for 30 min at 2500 × g and 4 °C. To achieve reduction of disulfide bridges, 2 µl of 1 M dithiothreitol (DTT) (Thermo Fisher Scientific, no. 20291, Rockford, IL, USA) was added to the samples and incubated on a rotating shaker for 60 min at 37 °C. Free cysteine residues were alkylated by adding 500 mM iodoacetamide (IAA) (Thermo Fisher Scientific, no. 90034, Rockford, IL, USA) to each sample vial, followed by incubation on the shaker for 30 min at 27 °C in the dark. The samples were then diluted with 414 µl (50 mM) TEAB to quench the reaction.

In-solution digestion was performed using 15 µl (30:1 protein:protease w/w ratio) Trypsin/Lys-C Protease Mix, MS Grade (Thermo Fisher Scientific, no. A40009, Rockford, IL, USA), overnight on a shaker at 37 °C. Protein digestion was stopped by adding 25% trifluoroacetic acid (TFA) (Thermo Fisher Scientific, no. 28904, Rockford, IL, USA). To eliminate impurities and perform desalting Pierce C18 Spin Tips & Columns (Thermo Fisher Scientific, no. 84850, Rockford, IL, USA) were used according to the manufacturer’s protocol.

### LC–MS/MS analysis

The purified peptides obtained from 30 µg of total proteome samples were rapidly dried using a concentrator plus speed vacuum system (Eppendorf, Hamburg, Germany) and subsequently reconstituted with a solution containing 0.1% TFA in a 5% acetonitrile (ACN) in distilled water for LC–MS analysis. Injection: 1 µl of each sample. Nano LC: UltiMate™ 3000 RSLCnano System (Dionex, Thermo Fisher Scientific, Sunnyvale, CA, USA); MS/MS system: Orbitrap Q Exactive Plus™ instrument (Thermo Fisher Scientific, Rockford, IL, USA). The peptide samples were pre-concentrated using a µ-pre-column, 300 µm ID, 5 µm particle size, and 100 Å pore size (Thermo Fisher Scientific, Rockford, IL, USA). Separation was performed using a 50-cm Acclaim PepMap™ 100 C18 column 50 cm × 75 µm, 2 µm (Thermo Fisher Scientific, Rockford, IL, USA). Flow rate 300 nL/min; mobile phase A: ultrapure water with 0.1% (v/v) formic acid); mobile phase 80% acetonitrile with 0.08% (v/v) formic acid); gradient: from 3 to 40% mobile phase B over 77 min and from 40 to 95% in 2 min, followed by a washing step at 95% B for 17 min. The MS Orbitrap Q Exactive Plus™ instrument (Thermo Fisher Scientific, Rockford, IL, USA) was operated in positive ion mode using data-dependent acquisition (DDA) with the following settings: spray voltage 2 kV; capillary temperature 275 °C; MS spectra: range of 375–1500 *m*/*z*, with a resolution of 70,000; AGC target 1e6; maximum ion accumulation time 50 ms. MS/MS spectra: Up to the top 15 precursor ions (charge states 2–6) were selected, with a resolution of 17,500. The dynamic exclusion time was set to 50 s and the isolation window to 2.0 *m*/*z*. For accuracy, 445.12003 *m*/*z* and 391.28429 *m*/*z* were used as lock masses.

### Computing resources used for MaxQuant software

The data underwent processing on a high-performance Linux computing cluster with CentOS 6.7 and CentOS 7 operating systems (Copyright 2024 The CentOS Project; https://www.centos.org/). The cluster consisted of seven computing nodes, each featuring either 24 cores at 2.6 GHz or 32 cores at 3.3 GHz, along with a maximum RAM capacity of 1 TB.

### LC–MS/MS acquired data analysis

The MaxQuant software version 2.0.3.1 was used to process and analyze the raw data (Tyanova et al. 2016a). Parameter grouping was specified as one given that we only utilized one single experiment of label-free data set. Distinct experiment labels were assigned to individual replicates to facilitate accurate normalizations. For the group specific tab, parameters were applied as the following: The type was set to “standard” for LFQ (label-free quantification); multiplicity was set to “1” (meaning no isotopic labeling was used); define “variable modifications” (oxidation (M), acetyl protein N-term); define “set modifications” (carbamidomethyl (C)); define digestion mode to “trypsin/P” and set max missed cleavages to “2”; set LFQ minimum ratio count to 2 and normalization type to “classic”. Identification was conducted using databases from both *Cricetulus griseus* (58 083 entries, UniProtKB id:10029, downloaded on 12 April 2023) and *Mus musculus* (88,534 entries, UniProtKB id:10090, downloaded on 12 April 2023) since Chinese hamster sequences possess the potential to encompass the entire mouse genome, despite the occurrence of intricate chromosomal rearrangements (Brinkrolf et al. 2013). The mass spectrometry proteomics data have been deposited to the ProteomeXchange Consortium via the PRIDE (Perez-Riverol et al. 2022) partner repository with the dataset identifier PXD049297.

The statistical workflow was conducted using the Perseus platform version 2.0.6.0 in strict adherence to the comprehensive guidelines provided in the following papers by its creators (Tyanova and Cox 2018; Tyanova et al. 2016b). We filtered the “proteinGroups.txt” file to eliminate entries annotated as “only identified by site,” “reverse,” and “potential contaminants.” Subsequently, the data matrix in the designated columns was subjected to a logarithmic transformation using a base-2 logarithm (log2). To systematically organize the conditions compared in this quantitative analysis, we categorized all the column data into three groups. This approach aimed to streamline comparisons across the cell lines. These groups were defined as 2G12, 353/11, and 353/11_TM.

To enhance the representation of each protein, additional entries were appended. These entries included Gene Ontology (GO) terms, Reactome, Gene Set Enrichment Analysis (GSEA), Kyoto Encyclopedia of Genes and Genomes (KEGG), mammalian protein complexes database (CORUM), Protein Families Database (Pfam), and Mouse Genome Identifier (MGI). Both *C. griseus* and *M. musculus* databases were utilized to maximize the annotated percentage.

To ensure data integrity and reliability, rows in the dataset were filtered to retain only those with a minimum of three valid values total, thus minimizing the presence of “NaN” (non-assigned number) values. Subsequently, each protein ID was complemented by incorporating updated gene annotations into new columns, ensuring the inclusion of the latest information for each entry. This annotation step was crucial for subsequent analyses, particularly in the calculation of enriched terms, as it facilitated the identification of relevant proteins.

A statistical analysis was performed to evaluate the significance of the observed changes in protein expression values. This involved the implementation of a two-sample *t* test, with the selection of values determined by a permutation-based false discovery rate (FDR). In order to account for multiple hypotheses, a significant threshold with a *q*-value of ≤ 0.05, determined through permutation-based FDR calculation, was employed. In this analysis, we determined the statistical significance of the observed differences between the groups 2G12 vs. 353/11 and 353/11_TM vs. 353/11, respectively. Relevant parameters, with S0 = 0 representing the threshold for fold difference in measurements, were taken into account during the evaluation process.

### topGO enrichment analysis of significant proteins

In order to conduct functional analysis on proteins and identify significant associated Gene Ontology (GO) terms, the topGO package was utilized. The GO term enrichment was calculated using the R library topGO v2.52.0 (Alexa and Rahnenfuhrer 2023). The list of all detected protein coding genes was defined as background and all differentially expressed protein coding genes with a *q*-value ≤ 0.05 as candidate genes. The GO terms corresponding to each gene were extracted from the *M. musculus*_gene_ensemble dataset of the ensemble release 110 (Martin et al. 2022). Significantly enriched GO terms have been determined using the weight 01 algorithm with Fisher’s exact test as test statistic. The R script for GO enrichment is available on GitHub: https://github.com/FLsandell.

### Functional enrichment analysis (by using DAVID software)

To provide additional information and enhance the clarity of our data, another biological analysis was performed using Database for Annotation, Visualization, and Integrated Discovery (Huang da et al. 2009; Sherman et al. 2022) (DAVID version 2021, https://david.ncifcrf.gov/tools.jsp). Mouse Genome Identifier (MGI) accessions or official Gene Symbols served as identifiers for each protein. Upon identifying enriched terms, a Benjamini–Hochberg false discovery rate (BH-FDR) *q*-value threshold of ≤ 0.05 was applied for data truncation. The terms meeting this criterion were considered significant and can be accessed in the supplementary data (ESM_1).

The visualization of these findings was accomplished using the Cytoscape (Shannon et al. 2003) version 3.10.0 with plug-ins from “EnrichmentMap Pipeline Collection” (Reimand et al. 2019). Parameters in the EnrichmentMap 3.3.6 were tuned as suggested by the creators with a *q*-value cutoff = 0.05 and overlap coefficient cutoff = 0.6. AutoAnnotate 1.4.1 was used as explained on its creators paper (Kucera et al. 2016).

### STRING protein–protein interactions

To predict known protein–protein interactions (PPI), we used the biological database STRING (https://string-db.org/) as outlined by Szklarczyk et al. (2023). Twenty-five significant proteins found in the GO terms (DAVID) from the 353/11_TM vs. 353/11 treated cells were used in calculations. To enhance the reliability of known or predicted protein interactions, these proteins were assigned the official *M. musculus* Official Gene Symbol. The minimum required confidence score of 0.4 was set for this analysis.

## Results

### Proteomics workflow and protein quantification

Samples were prepared for label-free quantification (LFQ) proteomics profiling with the goal of investigating differentially expressed proteins and uncovering associated signaling pathways. Our study involved a thorough comparative analysis of proteomic profiles from two different cell lines that express monoclonal antibodies (mAbs) at varying production levels. One cell line exhibited high production (353/11), whereas the other one (2G12) displayed low production. The data illustrating these differences are detailed in a previously published paper from our group, in which the naturally occurring 2G12 mAb (low producer) is compared to the corresponding germline-derived cognate 353/11 mAb (high producer) (Schwaigerlehner et al. 2019). Additionally, we examined the effects of tunicamycin (TM) addition, a compound known for its ability to inhibit protein glycosylation by targeting the initial phase of the lipid-linked pathway, which is described as TM-induced ER stress (Elbein 1987). Throughout the 18 samples analyzed (6 for each cell line group) using LFQ in MaxQuant, we successfully identified and analyzed a total of 2662 proteins using 19,958 unique and shared peptides, as outlined in the “Materials and methods” section.

To ensure the quality of the data, we implemented a filtering process that required valid values for proteins in at least three samples total. As a result, the dataset had a minimal number of missing values, and no imputation was performed for these instances. This yielded a final set of 1757 proteins for further analysis. Supplementary information, comprising additional details extracted from the analyzed data such as Uniprot identifiers, gene names, log2-transformed LFQ intensity values, MS/MS count, and associated statistical calculations for the 1757 proteins, is available in (ESM_1, Table S1). Moreover, Fig. 2 furnishes a depiction of the entire proteome analysis, elucidating its principal steps.

To evaluate the presence of significant differences in protein expression, we performed a two-sample *t* test using permutation-based false discovery rate (FDR) with a threshold of *q* ≤ 0.05. This analysis is being conducted between the groups 2G12 vs. 353/11 and 353/11_TM vs. 353/11, respectively. Further analysis revealed that we identified 100 significant hits between the 2G12 (low producer) and 353/11 (high producer) groups. Additionally, we found 31 significant hits between the 353/11_TM (high producer treated with TM) and 353/11 (high producer) groups (ESM_1, Table S1_1). Only two proteins were identified to be shared across the comparisons, resulting in a total of 129 significant proteins as detailed in (ESM_1, Table S1_1).

### Identification of significant changes in protein levels

**Fig. 3:**
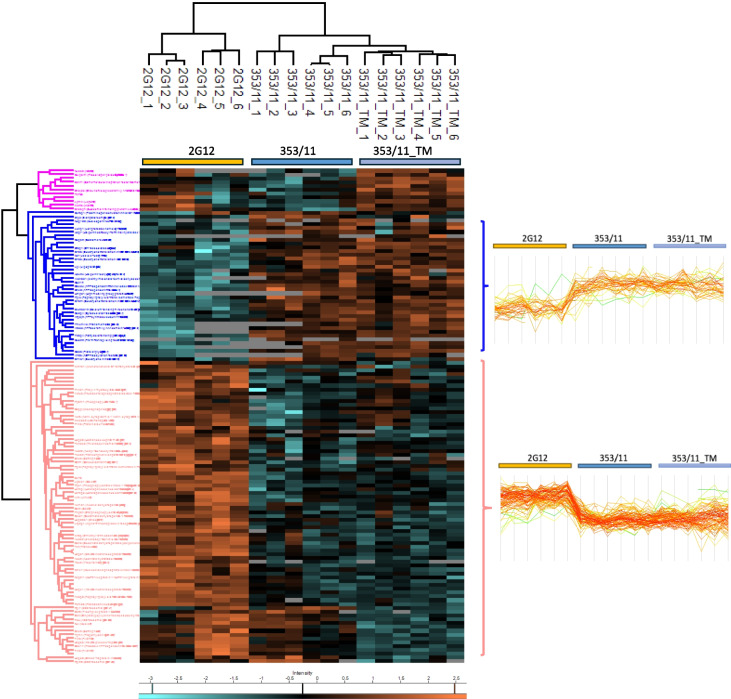
The heatmap illustrates significant proteins (permutation-based FDR *q* ≤ 0.05) in the comparisons of 2G12 vs. 353/11 and 353/11_TM vs. 353/11. Proteins with high expression are represented in orange, while those with low expression are displayed in blue. The notations “2G12,” “353/11,” and “353/11_TM” correspond to each cell line group, with appended numbers separated by “underscore” character representing replicates within each respective group. Two main clusters are indicated on the right as profile plots with their expression levels

To elucidate the disparities among the 129 identified proteins (ESM_1, Table S1_1) from the two-sample *t* test for each group comparison, we conducted an auxiliary analysis using hierarchical clustering. The data were normalized using Z-score normalization and visualized it as a heatmap (Fig. 3) showing the level of abundance-intensity expression per protein (high level, orange; low level, blue; “NaN” value, gray). Clustering of the samples revealed the formation of three clusters (pink, blue, and red) based on varying abundance-intensity levels of proteins across our three cell line groups (2G12, 353/11, 353/11_TM, respectively). Within the identified clusters, 39 proteins (blue cluster) exhibited downregulation in 2G12 compared to both 353/11 and 353/11_TM. Conversely, 79 proteins (red cluster) displayed upregulation in 2G12 relative to 353/11 and 353/11_TM. Meanwhile, the final cluster (pink cluster), comprising 11 proteins, demonstrated upregulation in 353/11_TM compared to 353/11, with a simultaneous upregulation in 2G12 compared to 353/11.

**Fig. 4:**
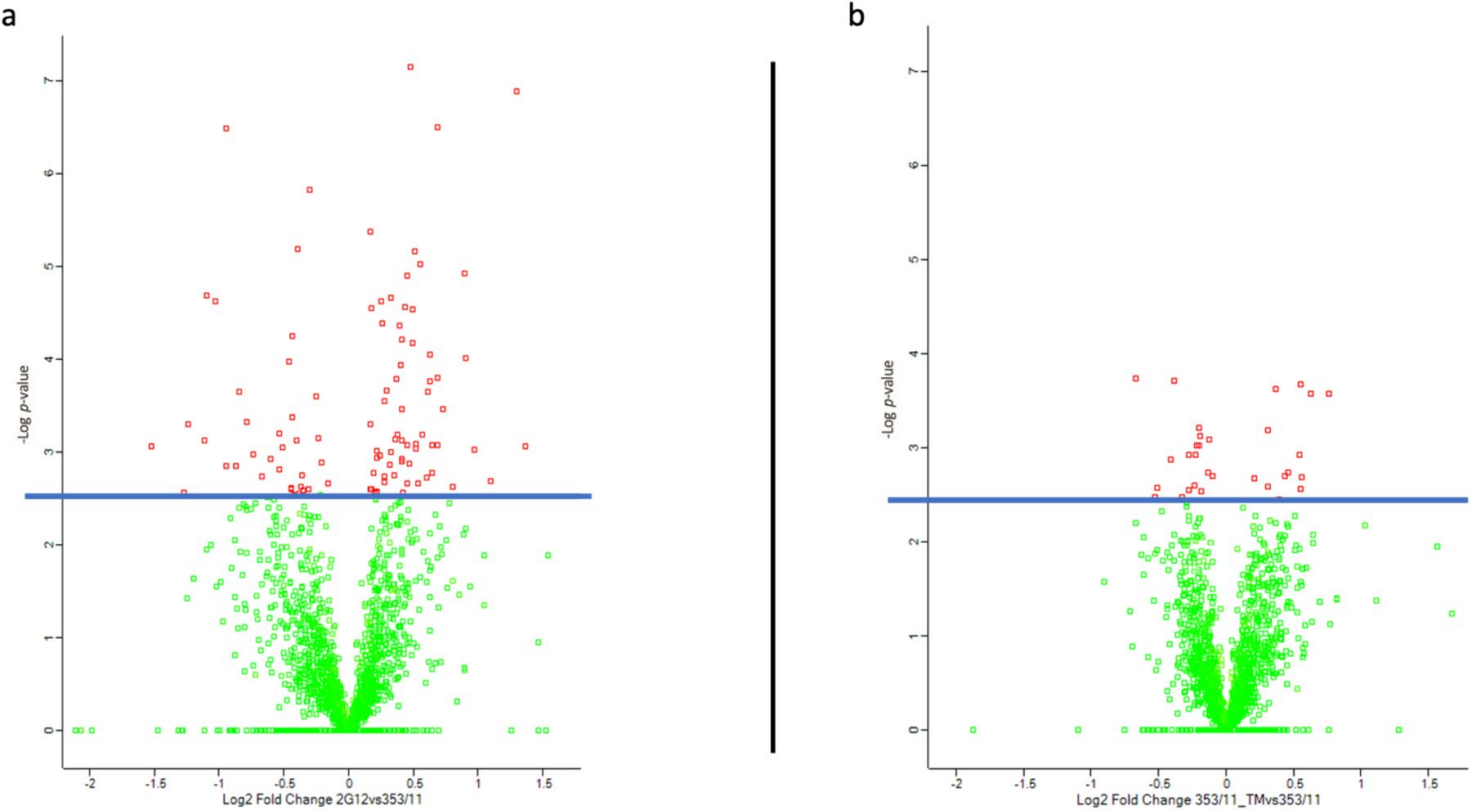
Volcano plots **a** and **b** depict fold changes of proteins adjusted for multiple testing using a permutation-based FDR, with *q* ≤ 0.05. The blue line demonstrates the threshold for a valid *q*-value. On the *X*-axis, the log2 fold change is shown between the groups **a** 2G12 vs. 353/11 and **b** 353/11_TM vs. 353/11, respectively. The *Y*-axis portrays − Log10 *p*-values, adhering to the corresponding *q*-value after correcting for multiple hypotheses. Notably, proteins colored in red exhibit *q* ≤ 0.05

To visualize the significant proteins used for the evaluations in Perseus software, a volcano plot was employed. This plot facilitated the clear representation of proteins that exhibited statistical significance in the analysis (Fig. 4). A threshold of *q* ≤ 0.05 that corresponds to its − Log10 *p*-values is used as the draw line between significant (shown in red) and non-significant values (shown in green). In Fig. 4 a and b, the 100 proteins emerging as significant in the comparisons between 2G12 and 353/11 are shown, whereas in the other comparison between 353/11_TM and 353/11, only 31 proteins demonstrate significance. Notably, only two proteins are found to be common between these two comparisons (refer to ESM_1, Table S1_1).

### Top 10 upregulated/downregulated proteins

Using the list of significant proteins obtained from the current two-sample *t* test (with a permutation-based FDR *q* ≤ 0.05) conducted between the groups 2G12 vs. 353/11 and 353/11_TM vs. 353/11, we have narrowed down the selection. Our attention is directed towards proteins exhibiting the top 10 highest mean log2 difference change. Table 1 and Table 2 present the downregulated and upregulated proteins, respectively, in 2G12 when compared to 353/11.

**Table 1:** Top 10 proteins significantly downregulated (*q* ≤ 0.05) in 2G12 (2G12 vs. 353/11)

Rank	− Log *p*-value	*q*-value	Log2 fold change	Protein	Gene name
1	3.06	0.01	− 1.51	Very-long-chain 3-oxoacyl-CoA reductase: involved in endoplasmic reticulum-bound enzymatic process	*Hsd17b12*
2	2.56	0.04	− 1.26	Protein disulfide isomerase Creld1: promotes the localization of acetylcholine receptors	*Creld1*
3	3.30	0.01	− 1.23	Methanethiol oxidase: involved in intra-Golgi protein transport	*Selenbp1*
4	3.12	0.01	− 1.11	High mobility group protein HMG-I/HMG-Y: involved in the transcription regulation of genes	*Hmga1*
5	4.69	0.00	− 1.09	Calcium-binding mitochondrial carrier protein Aralar1	*Slc25a12*
6	4.62	0.00	− 1.03	Fatty acid-binding protein, adipocyte	*Fabp4*
7	2.85	0.02	− 0.94	Cytoskeleton-associated protein 4	*Ckap4*
8	6.48	0.00	− 0.94	Platelet glycoprotein 4: plays a role in ER stress response	*Cd36*
9	2.85	0.02	− 0.87	Nucleoporin NUP188 homolog: RNA export from nucleus; protein import into nucleus	*Nup188*
10	3.64	0.00	− 0.84	ATPase family AAA domain-containing protein 3: may play an important role in mitochondrial protein synthesis	*Atad3*

**Table 2:** Top 10 proteins significantly upregulated (*q* ≤ 0.05) in 2G12 (2G12 vs. 353/11)

Rank	− Log *p*-value	*q*-value	Log2 fold change	Protein	Gene names
1	3.06	0.01	1.36	Cathepsin Z	*Ctsz*
2	6.88	0.00	1.30	Peptidyl-prolyl cis–trans isomerase FKBP9: PPIases accelerate the folding of proteins during protein synthesis	*Fkbp9*
3	2.69	0.04	1.09	Macrophage-capping protein	*Capg*
4	3.02	0.01	0.97	Sorting nexin-3: plays a role in protein transport between cellular compartments	*Snx3*
5	4.92	0.00	0.89	10 kDa heat shock protein, mitochondrial: together with Hsp60, facilitates the correct folding of imported proteins	*Hspe1*
6	2.62	0.04	0.80	Prolyl 4-hydroxylase subunit alpha-1	*P4ha1*
7	3.45	0.01	0.73	Phosphatidylethanolamine-binding protein 1: inhibitor of MEK phosphorylation	*Pebp1*
8	6.50	0.00	0.69	Galectin-1: plays a role in regulating apoptosis, cell proliferation and cell differentiation	*Lgals1*
9	3.07	0.01	0.69	Y-box-binding protein 3: may have a role in translation repression	*Ybx3*
10	3.80	0.00	0.68	Fructose-bisphosphate aldolase; fructose-bisphosphate aldolase A: may also function as scaffolding protein	*Aldoa*

In the other comparison, 353/11_TM vs. 353/11, Table 3 and Table 4 delineate the downregulation and upregulation of proteins in TM-treated samples as opposed to their non-treated counterparts.

**Table 3:** Top 10 proteins significantly down regulated (*q* ≤ 0.05) in 353/11_TM (353/11_TM vs. 353/11)

Rank	− Log *p*-value	*q*-value	Log2 fold change	Protein	Gene names
1	3.73	0.00	− 0.66	T-complex protein 1 subunit theta: assists the folding of proteins upon ATP hydrolysis	*Cct8*
2	2.47	0.03	− 0.52	60S ribosomal protein L28: component of the large ribosomal subunit	*Rpl28*
3	2.57	0.04	− 0.51	NADH-ubiquinone oxidoreductase 75 kDa subunit, mitochondrial: core subunit of the mitochondrial membrane	*Ndufs1*
4	2.88	0.00	− 0.41	Cornifin-A: cross-linked envelope protein of keratinocytes	*Sprr1a*
5	3.71	0.00	− 0.38	PDZ and LIM domain protein 5	*Pdlim5*
6	2.47	0.03	− 0.32	Plasminogen activator inhibitor 1 RNA-binding protein: may play a role in the regulation of mRNA stability	*Serbp1*
7	2.92	0.00	− 0.27	Dihydrolipoyllysine-residue succinyltransferase component of 2-oxoglutarate dehydrogenase complex, mitochondrial	*Dlst*
8	2.54	0.03	− 0.27	40S ribosomal protein S30: belongs to the ubiquitin family	*Fau;fau*
9	2.59	0.04	− 0.23	Stress-70 protein, mitochondrial: may play a role in the control of cell proliferation	*Hspa9*
10	2.92	0.00	− 0.22	Probable ATP-dependent RNA helicase DDX17: Involved in multiple cellular processes	*Ddx17*

**Table 4:** Top 10 proteins significantly up regulated (*q* ≤ 0.05) in 353/11_TM, (353/11_TM vs. 353/11)

Rank	− Log *p*-value	*q*-value	Log2 fold change	Protein	Gene names
1	3.57	0.00	0.75	Barrier-to-autointegration factor; barrier-to-autointegration factor, N-terminally processed	*Banf1*
2	3.56	0.00	0.62	Legumain: plays a role in the regulation of cell proliferation via its role in EGFR degradation	*Lgmn*
3	2.68	0.03	0.55	Nedd8: ubiquitin-like protein which plays an important role in cell cycle control	*Nedd8*
4	2.56	0.03	0.55	DnaJ homolog subfamily A member 3, mitochondrial	*Dnaja3*
5	3.66	0.00	0.55		*I79_000465*
6	2.74	0.01	0.46	Procollagen galactosyltransferase 1: involved in the biosynthesis of collagen type IV	*Colgalt1*
7	2.70	0.03	0.43	Matrin 3: may play a role in nuclear retention of defective RNAs	*Matr3*
8	2.44	0.04	0.39	Peroxiredoxin-5: plays a role in cell protection against oxidative stress by detoxifying peroxides	*Prdx5*
9	3.62	0.00	0.36	SH3 domain-binding glutamic acid–rich-like protein	*Sh3bgrl*
10	2.59	0.04	0.31	26S proteasome regulatory subunit 6A: involved in the maintenance of protein homeostasis by removing misfolded or damaged proteins	*Psmc3*

The magnitude of fold change was slightly greater in low producer vs. high producer comparison than in TM-treated samples. In this list, the two proteins namely (1) Sh3bgrl and (2) Hsp90b1 identified as common between the two comparisons did not surpass the threshold for inclusion among the top 10 proteins. For further statistical information regarding each protein, refer to the supplementary information in (ESM_1, Table S1_1).

### topGO enrichment analysis on proteins

topGO was used for providing insights into the biological functions and processes associated with all significant proteins from both comparisons (2G12 vs. 353/11 and 353/11_TM vs. 353/11). To ensure greater stringency and accuracy in the results, the cutoff value was set at *q* < 0.02. Within (ESM_1, Tables S2 and S3), it was evident that among the terms identified in both 2G12 vs. 353/11 and 353/11_TM vs. 353/11, four were found to be common. These terms include the following: GO_MF, ATP-dependent protein folding chaperone; GO_CC, endoplasmic reticulum lumen; GO_BP, negative regulation of apoptotic process; and GO_BP, proteolysis involved in protein catabolic process.

This suggests that TM-treated cells, compared to low and high producer cell lines, represent an overlapping cellular response to proteins expression levels of the low producer 2G12.

### Enrichment analysis (by using DAVID software)

**Fig. 5:**
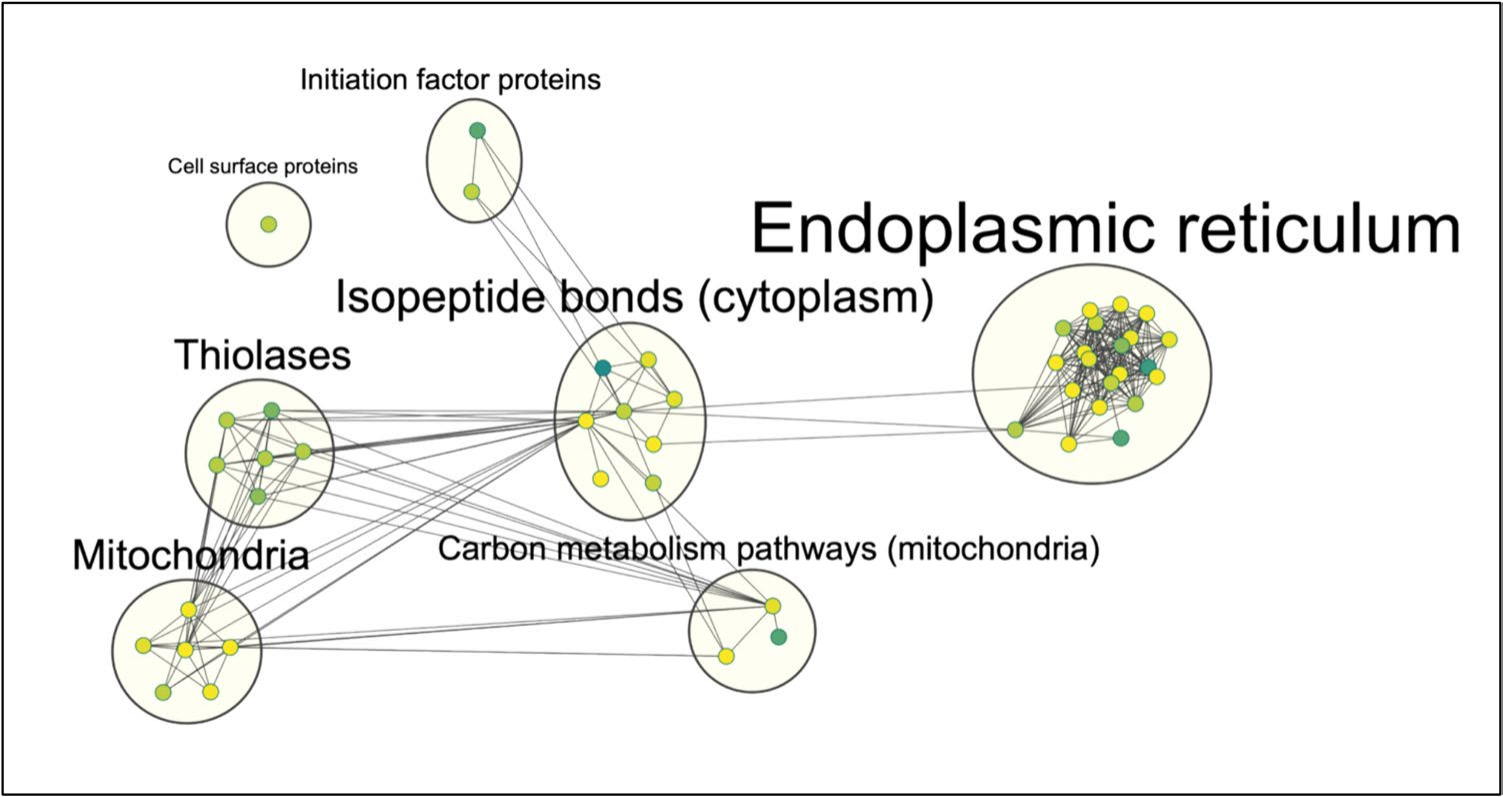
An enrichment chart depicting a visual representation of a pathway enrichment analysis (top significant terms (BH-FDR, *q*-value 0.05) resulting from DAVID functional enrichment analysis listed in Table 5 between 2G12 and 353/11, where nodes symbolize pathways, and the edges signify the intercommunication between interconnected pathways. The parameters for the cluster creation are as following: *q*-value cutoff 0.05 with an overlap coefficient cutoff 0.6. Nodes represent protein sets corresponding to one enriched term. Terms which have an overlap score 0.6 cluster together

**Fig. 6:**
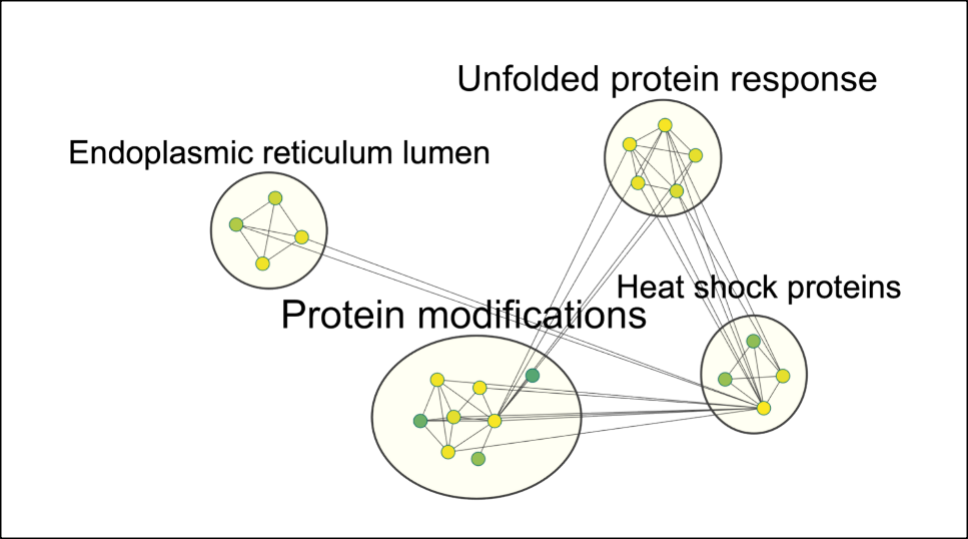
An enrichment chart depicting a visual representation of a pathway enrichment analysis (top significant terms (BH-FDR, *q*-value 0.05) resulting from DAVID functional enrichment analysis listed in Table 6 between 353/11_TM vs. 353/11), where nodes symbolize pathways, and the edges signify the intercommunication between interconnected pathways. The parameters for the cluster creation are as following: *q*-value cutoff 0.05 with an overlap coefficient cutoff 0.6. Nodes represent protein sets corresponding to one enriched term. Terms which have an overlap score 0.6 cluster together

Additionally, the identification of significantly over-represented functional terms within the lists of all significant proteins between two comparisons was conducted using DAVID (ESM_1, Tables S4 and S5). Only functional terms with *q*-value smaller than 0.05 are shown in Table 5 and Table 6. Furthermore, using the Cytoscape (Shannon et al. 2003) version 3.10.0 with plug-ins from “EnrichmentMap Pipeline Collection” (Reimand et al. 2019), we were able to organize and visualize the terms in clusters as depicted in Figs. 5 and 6.

**Table 5:** Functional enrichment analysis DAVID for 2G12 vs. 353/11

**Category**	**Term**	**% proteins associated with the term**	***p*-value**	**Benj. Hoch. FDR**
**Significant terms in connection with mitochondria**				
GOTERM_CC_DIRECT	GO:0005739 ~ mitochondrion	32.47	0.0000	0.0000
UP_KW_DOMAIN	KW-0809 ~ transit peptide	14.29	0.0000	0.0001
UP_KW_CELLULAR_COMPONENT	KW-0496 ~ mitochondrion	22.08	0.0000	0.0007
GOTERM_CC_DIRECT	GO:0005759 ~ mitochondrial matrix	10.39	0.0000	0.0013
UP_SEQ_FEATURE	TRANSIT: mitochondrion	12.99	0.0001	0.0052
GOTERM_CC_DIRECT	GO:0005743 ~ mitochondrial inner membrane	11.69	0.0006	0.0128
**Significant terms in connection with endoplasmic reticulum**				
GOTERM_BP_DIRECT	GO:0006457 ~ protein folding	12.99	0.0000	0.0000
UP_SEQ_FEATURE	MOTIF: prevents secretion from ER	10.39	0.0000	0.0000
UP_KW_MOLECULAR_FUNCTION	KW-0413 ~ isomerase	12.99	0.0000	0.0000
GOTERM_MF_DIRECT	GO:0016853 ~ isomerase activity	12.99	0.0000	0.0000
GOTERM_CC_DIRECT	GO:0005788 ~ endoplasmic reticulum lumen	10.39	0.0000	0.0000
GOTERM_MF_DIRECT	GO:0003756 ~ protein disulfide isomerase activity	6.49	0.0000	0.0001
UP_KW_DOMAIN	KW-0676 ~ redox-active center	6.49	0.0000	0.0002
INTERPRO	IPR005788: disulfide isomerase	5.19	0.0000	0.0002
UP_SEQ_FEATURE	DOMAIN: thioredoxin 1	5.19	0.0000	0.0004
UP_SEQ_FEATURE	DOMAIN: thioredoxin 2	5.19	0.0000	0.0004
UP_SEQ_FEATURE	DISULFID: redox-active	6.49	0.0000	0.0025
GOTERM_CC_DIRECT	GO:0005783 ~ endoplasmic reticulum	23.38	0.0001	0.0030
INTERPRO	IPR017937: thioredoxin, conserved site	5.19	0.0000	0.0039
GOTERM_MF_DIRECT	GO:0015035 ~ protein disulfide oxidoreductase activity	5.19	0.0001	0.0097
GOTERM_CC_DIRECT	GO:0042470 ~ melanosome	6.49	0.0005	0.0120
GOTERM_CC_DIRECT	GO:0034663 ~ endoplasmic reticulum chaperone complex	3.90	0.0008	0.0148
INTERPRO	IPR013766: thioredoxin domain	5.19	0.0004	0.0147
UP_KW_CELLULAR_COMPONENT	KW-0256 ~ endoplasmic reticulum	18.18	0.0011	0.0149
UP_SEQ_FEATURE	DOMAIN: thioredoxin	5.19	0.0005	0.0250
UP_KW_MOLECULAR_FUNCTION	KW-0143 ~ chaperone	7.79	0.0043	0.0367
KEGG_PATHWAY	mmu04141: protein processing in endoplasmic reticulum	7.79	0.0010	0.0416
**Significant terms in connection with isopeptide bonds (cytoplasm)**				
UP_KW_PTM	KW-0007 ~ acetylation	74.03	0.0000	0
GOTERM_CC_DIRECT	GO:0043209 ~ myelin sheath	15.58	0.0000	0
GOTERM_CC_DIRECT	GO:0005829 ~ cytosol	45.45	0.0000	0.0001
GOTERM_CC_DIRECT	GO:0005737 ~ cytoplasm	57.14	0.0001	0.0043
UP_KW_PTM	KW-1017 ~ isopeptide bond	25.97	0.0006	0.0048
GOTERM_CC_DIRECT	GO:0032991 ~ macromolecular complex	15.58	0.0005	0.012
UP_KW_PTM	KW-0597 ~ phosphoprotein	72.73	0.0023	0.0122
**Significant terms in connection with thiolases**				
INTERPRO	IPR020615: thiolase, acyl-enzyme intermediate active site	3.90	0.0003	0.0147
INTERPRO	IPR020617: thiolase, C-terminal	3.90	0.0003	0.0147
INTERPRO	IPR020613: thiolase, conserved site	3.90	0.0003	0.0147
INTERPRO	IPR020616: thiolase, N-terminal	3.90	0.0004	0.0147
UP_SEQ_FEATURE	DOMAIN: thiolase N-terminal	3.90	0.0004	0.023
INTERPRO	IPR016039: thiolase-like	3.90	0.0009	0.0277
**Significant terms in connection with metabolic pathways (mitochondria)**				
KEGG_PATHWAY	mmu01200: carbon metabolism	11.69	0.0000	0
KEGG_PATHWAY	mmu01100: metabolic pathways	24.68	0.0001	0.0048
UP_KW_MOLECULAR_FUNCTION	KW-0560 ~ oxidoreductase	12.99	0.0040	0.0367
**Significant terms in connection with initiation factor proteins**				
UP_KW_BIOLOGICAL_PROCESS	KW-0648 ~ protein biosynthesis	7.79	0.0002	0.0124
UP_KW_MOLECULAR_FUNCTION	KW-0396 ~ initiation factor	5.19	0.0020	0.0348
**Significant terms in connection with cell surface proteins**				
GOTERM_CC_DIRECT	GO:0009986 ~ cell surface	14.29	0.0006	0.0128

**Table 6:** Functional enrichment analysis DAVID for 353/11_TM vs. 353/11

**Category**	**Term**	**% proteins associated with the term**	***p*-value**	**Benj. Hoch. FDR**
**Significant terms in connection with unfolded protein response**				
GOTERM_MF_DIRECT	GO:0051082 ~ unfolded protein binding	19.23	0.0000	0.0009
GOTERM_MF_DIRECT	GO:0044183 ~ protein binding involved in protein folding	15.38	0.0000	0.0022
GOTERM_BP_DIRECT	GO:0006457 ~ protein folding	19.23	0.0000	0.0032
GOTERM_MF_DIRECT	GO:0016887 ~ ATPase activity	23.08	0.0002	0.0047
UP_KW_MOLECULAR_FUNCTION	KW-0143 ~ chaperone	19.23	0.0004	0.0069
**Significant terms in connection with protein modifications (cytoplasm/cytosol)**				
GOTERM_CC_DIRECT	GO:0005737 ~ cytoplasm	80.77	0.0000	0.0008
GOTERM_CC_DIRECT	GO:0005829 ~ cytosol	61.54	0.0000	0.0012
UP_SEQ_FEATURE	CROSSLNK: glycyl lysine isopeptide (Lys-Gly) (interchain with G-Cter in SUMO2); alternate	23.08	0.0000	0.0020
UP_SEQ_FEATURE	CROSSLNK: glycyl lysine iso-peptide (Lys-Gly) (interchain with G-Cter in SUMO1); alternate	19.23	0.0000	0.0020
UP_KW_PTM	KW-1017 ~ isopeptide bond	38.46	0.0008	0.0050
GOTERM_CC_DIRECT	GO:1,990,904 ~ ribonucleoprotein complex	19.23	0.0012	0.0223
UP_SEQ_FEATURE	CROSSLNK: glycyl lysine isopeptide (Lys-Gly) (interchain with G-Cter in SUMO2)	26.92	0.0009	0.0307
GOTERM_CC_DIRECT	GO:0048471 ~ perinuclear region of cytoplasm	23.08	0.0026	0.0359
**Significant terms in connection with heat shock proteins**				
UP_KW_PTM	KW-0007 ~ acetylation	76.92	0.0000	0.0000
GOTERM_MF_DIRECT	GO:0031072 ~ heat shock protein binding	15.38	0.0001	0.0047
UP_KW_DOMAIN	KW-0809 ~ transit peptide	19.23	0.0024	0.0214
GOTERM_CC_DIRECT	GO:0043209 ~ myelin sheath	15.38	0.0015	0.0233
**Significant terms in connection with endoplasmic reticulum lumen**				
UP_SEQ_FEATURE	MOTIF: prevents secretion from ER	15.38	0.0001	0.0025
GOTERM_CC_DIRECT	GO:0034663 ~ endoplasmic reticulum chaperone complex	11.54	0.0001	0.0032
GOTERM_CC_DIRECT	GO:0005788 ~ endoplasmic reticulum lumen	15.38	0.0004	0.0100
GOTERM_CC_DIRECT	GO:0005790 ~ smooth endoplasmic reticulum	11.54	0.0007	0.0162

This analysis revealed that the most significant terms in 2G12 vs. 353/11 were found to be related to (I) mitochondria, (II) endoplasmic reticulum, (III) isopeptide bonds (cytoplasm), (IV) thiolases, (V) metabolomic pathways (mitochondria), (VI) initiation factor proteins, and (VII) cell surface proteins (Table 5; Fig. 5). Interestingly, in the comparison between 2G12 (low producer) and 353/11 (high producer), prominent terms related to mitochondria were observed, indicating potential alterations in this essential cellular organelle, as it is the center of oxidative metabolism for both sample groups (Table 5; Fig. 5).

In the other comparison 353/11_TM vs. 353/11, the most significant biological terms were found to be associated with (I) unfolded protein response, (II) protein modifications (cytoplasm/cytosol), (III) heat shock proteins, and (IV) endoplasmic reticulum lumen (Table 6; Fig. 6).

In the comparison between 353/11_TM (high producer treated with TM) and 353/11 (high producer), we found terms related to UPR which is in correlation with the effect that TM has on protein folding (Table 6; Fig. 6).

### Similarities obtained between the two enrichment platforms: topGO and DAVID

Observed variations and similarities were based on two influencing factors: the distinct protein expression profiles and the TM treatment. A total of 36 proteins were downregulated, and 64 were upregulated when comparing low producer vs. high producer. In the comparison between TM-treated high producer vs. high producer, 18 proteins were identified as downregulated, while 13 were upregulated.

Both analytical platforms topGO and DAVID focused on the two protein comparisons: 2G12 vs. 353/11 and 353/11_TM vs. 353/11. The consistent identification of enriched terms, such as endoplasmic reticulum, ATP-dependent protein folding chaperones, negative regulation of apoptotic processes, and proteolysis, underlined the relevance across multiple analysis (supplementary material, ESM_1).

Cytoscape visualization apps were employed to enhance the representation of enriched terms using data obtained from DAVID. The visualizations depicted biological associations, showcasing the impact on mitochondria and endoplasmic reticulum, along with the intricate interplay of protein folding and the unfolded protein response (UPR) (Figs. 5 and 6). In-depth analyses of low producer vs. high producer comparison, and TM-treated high producer vs. high producer, provided insights into the effects of treatments on cellular processes, particularly in the context of ER stress pathways and UPR (Tables 5 and 6; Figs. 5 and 6).

In the comparative analysis between 2G12 vs. 353/11, significant congruence was observed in major annotations derived from both DAVID and topGO. The meticulous categorization of annotations, which are associated with the endoplasmic reticulum, yielded the following results: GO:0140662 ~ ATP-dependent protein folding chaperone (topGO); GO:0005788 ~ endoplasmic reticulum lumen (topGO); GO:0006457 ~ protein folding (DAVID); MOTIF ~ prevents secretion from ER (DAVID); GO:0005788 ~ endoplasmic reticulum lumen (DAVID); GO:0005783 ~ endoplasmic reticulum (DAVID); KW-0648 ~ protein biosynthesis (DAVID); KW-0256 ~ endoplasmic reticulum (DAVID); and mmu04141 ~ protein processing in endoplasmic reticulum (DAVID).

In the comparative analysis of 353/11_TM versus 353/11, distinctive endoplasmic reticulum–associated terms emerged, underlining the nuanced molecular characteristics of the respective entities. Noteworthy annotations encompassed: GO:0140662 ~ ATP-dependent protein folding chaperone (topGO); GO:0051082 ~ unfolded protein binding(topGO); GO:0005788 ~ endoplasmic reticulum lumen (topGO); GO:0034663 ~ endoplasmic reticulum chaperone complex (topGO); GO:0006457 ~ protein folding (topGO); GO:0051082 ~ unfolded protein binding (DAVID); GO:0044183 ~ protein binding involved in protein folding (DAVID): MOTIF ~ prevents secretion from ER (DAVID); GO:0006457 ~ protein folding (DAVID); GO:0034663 ~ endoplasmic reticulum chaperone complex (DAVID); KW-0143 ~ chaperone (DAVID): GO:0005788 ~ endoplasmic reticulum lumen (DAVID); and GO:0005790 ~ smooth endoplasmic reticulum (DAVID). These annotations collectively underline the molecular nuances governing protein folding, chaperone activities, and endoplasmic reticulum dynamics within the context of the comparative analysis. The consistent identification of these annotations across two analytical platforms reinforces the reliability and significance of the findings, highlighting commonalities in endoplasmic reticulum–related processes.

The two analytical platform topGO and DAVID enrichment analyses collectively painted a detailed portrait of the functional landscape of proteins, revealing similarities, disparities, and potential regulatory mechanisms in response to different experimental conditions. These findings may lay the groundwork for a deeper understanding of cellular dynamics and pave the way for future investigations into the intricate world of protein signaling pathways.

### STRING analysis for proteins with enriched terms (353/11_TM vs. 353/11)

**Fig. 7:**
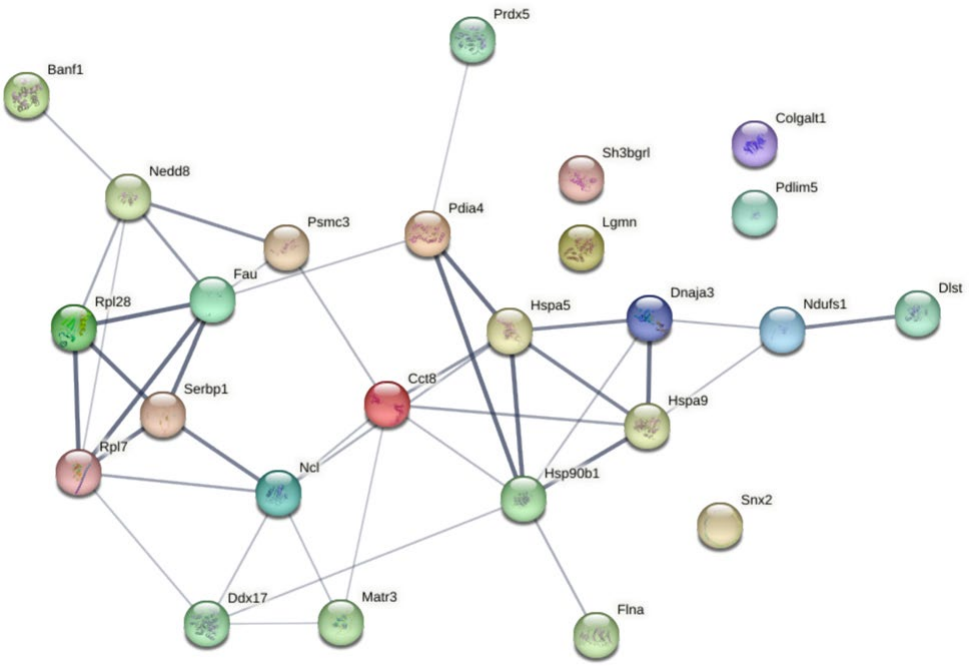
STRING functional protein enrichment from 353/11_TM comparison to 353/11. Proteins connected by a line have a confidence level between 0.400 (medium confidence) and 0.900 (highest confidence). Confidence level intensity and strength of data support between two proteins are indicated by the line thickness

The STRING functional enrichment analysis of differentially expressed proteins in the comparison between 353/11_TM (high producer treated with TM) and 353/11 (high producer) reveals enriched terms such as biological processes (GO): protein folding (Cct8, Hspa9, Hspa5, Hsp90b1, Dnaja3, Pdia4); molecular function (GO): unfolded protein binding (Hspa9, Hspa5, Hsp90b1, Dnaja3, Cct8); and cellular component (GO): endoplasmic reticulum (Hspa5, Hsp90b1, Pdia4) (ESM_1, Table S6). Additionally, a noteworthy observation is the identification of another cluster node of four proteins (Ncl; Dnaja3; Hspa9; Hsp90b1) which in another paper (Londono et al. 2012) identifies them to play a role in controlling cell proliferation and inhibiting apoptosis as part of the stress response (Fig. 7).

## Discussion

### Comparison of protein expression levels of low producer vs. high producer CHO cell lines

In our proteomics approach, distinct disparities emerged in the comparison of 2G12 (low producer) and 353/11 (high producer), demonstrating the most noteworthy abundance of significant proteins. This observation led to the identification of 100 proteins in total, thereby highlighting the differences in protein expression levels between these two cell line groups. Additionally, a higher number of enriched terms were exclusively observed in this comparison. Notably, we observed a pronounced increase in the upregulation of proteins associated with the protein folding mechanisms. This upregulation was particularly intriguing given that it was mostly correlated with the ER. Within this frame, our analysis identified a cohort of several proteins associated with the following enriched term “GOTERM_BP_DIRECT: protein folding.” These proteins include Fkbp9, Hspe1, Hspd1, Hspa8, Hsp90b1, Ppia, Ppic, P4hb, Pdia3, and Txndc5 (ESM_1, Table S4).

Sixty-four out of 100 significantly regulated proteins displayed upregulation in the low producer cell line compared to the high producer. The remaining 36 proteins displayed downregulation in low producer cell lines. One intriguing example was the Ppic protein, which demonstrated a minor downregulation. Ppic belongs to the peptidyl-prolyl cis–trans isomerase (PPIase) family and is known to facilitate protein folding acceleration (Friedman and Weissman 1991). Among the significant proteins in the low producer cell line, Hspa8 (heat shock protein 70 family) and Hsp90b1, both recognized components of the unfolded protein response (UPR) pathway (Wang et al. 2000) showed distinct increases in expression. Additionally, other proteins associated with the mitochondrial unfolded protein response (UPRmt), such as Hspd1 and Hspe1 (Munch and Harper 2016), exhibited upregulation as well. The upregulation of these proteins led us to hypothesize that the low producer cell line was experiencing a form of ER stress related to recombinant protein formation.

### Comparison of low producer vs. high producer and TM treated high producer vs. high producer

In both our cell line group comparisons, only two proteins were consistently present on the significant lists namely Sh3bgrl and Hsp90b1.

Sh3 domain-binding glutamic acid–rich-like protein (Sh3bgrl) displayed upregulation (log2 fold change of 0.41 and 0.37) in both comparisons, respectively. Sh3bgrl was recognized as a scaffold protein that participates in numerous protein–protein interactions (Cesareni et al. 2002). Its structure and sequence placed it within the homologous superfamily of thioredoxin (Yin et al. 2005). Recent research indicates that Sh3bgrl exerts inhibitory effects on cell proliferation and induces cell cycle arrest (Saleh et al. 2023). This implied that TM treatment affected the high producer cell line in a manner similar to the low producer cell line, and the elevated Sh3bgrl expression might correlate with suboptimal protein production by mildly affecting cell proliferation. Sh3bgrl was also predicted to participate in positive regulation of cytoplasmic translational initiation and proteasome-mediated ubiquitin-dependent protein catabolic processes (Zhang et al. 2021).

The second protein, heat shock protein 90 beta family member 1, was identified as significant in both of our comparisons. It is a well-established UPR protein, which is known to facilitate proteasome-dependent degradation (Marcu et al. 2002). Interestingly, it was not upregulated upon TM treatment; instead, it exhibited a low level of downregulation when compared to the non-treated cells. This unexpected result could be attributed to the brief 4-h TM treatment. This short time frame might have constrained the translation machinery’s capacity to synthesize these proteins.

A separate study conducted with neuroblastoma cells, revealed a similar pattern, supporting our data (Bull and Thiede 2012). In this study, cells were exposed to a higher concentration of around 8 μg/ml for a longer duration of 8 h, compared to our 4-h treatment with 1 μg/ml. The results demonstrated that the majority of quantified proteins remained unaltered following the 8-h TM treatment, which is similar to our obtained data. Specifically, 99.5% of the quantified proteins exhibited a fold change ratio of less than twofold when compared to control cells (Bull and Thiede 2012), aligning with the outcomes observed in our own investigation.

### Proteins related to mitochondrial UPR upon tunicamycin treatment

In the list of significantly regulated proteins (ESM_1, Table S1_1), we identified candidates that were activated by the mitochondrial UPR (UPRmt) (Gottlieb and Bernstein 2016). One example was Hspa9, a 70-kDa heat shock chaperone protein from the HSP70 family, which is predominantly found in mitochondria (Wadhwa et al. 1993) or endoplasmic reticulum (Ran et al. 2000). The Hspa9 protein is further involved in protein folding processes that occur in mitochondria, along with another mitochondrial chaperone, Hspa60 (Deocaris et al. 2006).

Another candidate protein was Dnaja3 (ESM_1, Table S1_1), a heat shock protein and chaperone belonging to the Hsp40 family, which is also a mitochondrial chaperone involved in protein transport and refolding, and additionally has been found to play a role in mtDNA maintenance (Ng et al. 2014). Dnaja3 has been shown to interact with the ATPase and stimulates the activity of HSp70 (Ng et al. 2014; Syken et al. 1999). Interestingly, the chaperone activity of these both proteins, as indicated by their expression levels, suggested that the mitochondrial unfolded protein response is induced by tunicamycin (TM) treatment. However, the Hspa9 protein levels remained slightly affected by TM treatment, while Dnaja3 was upregulated. These findings aligned with previous studies, which addressed an increase in Dnaja3 expression following TM treatment (Koo et al. 2012; Mungrue et al. 2009). Furthermore, elevated Dnaja3 protein level has also been associated with UPRmt as reported before (Beck et al. 2016). The presence of chaperones Hspa9 and Dnaja3 in our findings supported the concept of a Dnaja3-Hspa9 chaperone interaction system proposed in a previous study (Ng et al. 2014). The STRING functional protein enrichment analysis also suggested a link between UPR pathway proteins and proteins involved in UPRmt (ESM_1, Table S6) (Londono et al. 2012).

Our findings further reinforced the idea, as addressed before in multiple studies (Nargund et al. 2012; Tran and Van Aken 2020) that unfolded proteins, which are unable to be imported into mitochondria trigger the activation of the mitochondrial unfolded protein response (UPRmt) to maintain mitochondrial functions.

### Nedd8, Hspa5/Bip, Pdia4, and Lgmn protein expression levels in TM stress response

One of the proteins that exhibited significant upregulation following TM treatment was Nedd8 (*q*-value = 0.035; log2 fold change = 0.55). It is a ubiquitin-like protein that plays a crucial role in the regulation of the ubiquitin–proteasome system (UPS), which is essential for maintaining protein homeostasis (Soucy et al. 2010). The process is called neddylation and it is a type of posttranslational modifications (PTMs) in which target proteins are conjugated with ubiquitin like protein Need8 (Li et al. 2015). The input of these PTMs have been found to induce an effect by modulating protein functions on cell proliferation, signal transduction (Zou et al. 2018), and transcriptional activity (Xirodimas et al. 2004). In addition, neddylation has been found to play a crucial role in protecting ribosomal proteins from instability (Xirodimas et al. 2008).

Moreover, we considered another protein that was found to be regulated in response to accumulation of misfolded proteins in the ER, known as Hspa5/Bip (*q*-value = 0.014; log2 fold change =  − 0.12), belonging to the Hsp70 family. Hspa5 protein acts as an ER chaperone (Kampinga and Craig 2010) and is associated with UPR pathway (Bertolotti et al. 2000; Yoshida et al. 2001). Our data showed that Hspa5/Bip was not drastically downregulated and apparently unaffected by TM treatment, corroborating a previous study which demonstrated that ER stressor (TM) seemed not to alter the levels of UPR pathway proteins in a drastic way (Kim et al. 2018).

In addition to Hspa5/Bip, our study also identified Pdia4 (*q* = 0; log2 fold change =  − 0.19) representative of the UPR pathway. Pdia4, a highly abundant ER protein, plays a critical role in protein folding and assembly (Ferrari and Soling 1999), is involved in the response to ER stress (Dorner et al. 1990), and participates in the formation and isomerization of disulfide bridges (Maattanen et al. 2010). Contrary to other research findings (Komatsu et al. 2020), in our data, we did not observe an increase in Pdia4 protein levels upon TM treatment. This observation provides additional support for the hypothesis that the events within the UPR machinery components constitute an early response cascade preceding the full UPR activation, as previously reported (Merksamer et al. 2008). To provide extra information, it is worth noting that in a different study, Bip/Hspa5 and Pdia4 were found to cooperate and to interact with each other in the folding of antibodies in vitro (Mayer et al. 2000).

Furthermore, cysteine protease Legumain (Lgmn; *q*-value = 0, log2 fold change = 0.62) was found to be upregulated. In a previous study, it was observed that the treatment with TM enhances the secretion of Legumain proteins. This finding aligned with the protein expression levels measured in our study. However, despite the observed elevated protein expression, TM treatment there led to a decrease in Legumain activity (Lunde et al. 2017).

### Mild tunicamycin treatment influences protein translational process in ribosomes

Research conducted a decade ago (Steffen et al. 2012) supported the hypothesis that reduced translation is a distinct mechanism independent from the unfolded protein response (UPR). This mechanism acts as a protective measure against ER stress (Steffen et al. 2012). Our study was in line with this model, as it demonstrated that all significant ribosomal proteins (Rpl28, Rpl7, Fau) are downregulated after TM treatment (ESM_1, Table S1_1). This finding suggests that these proteins may be directly involved in the early response to ER stress.

In another study, it has been shown that TM treatment also repressed the transcription of ribosomal protein (RP) genes (Miyoshi et al. 2001). This also aligns with our hypothesis, as we observed a similar pattern in our case.

In summary, our study successfully quantified a total of 1757 proteins across three experimental groups. Notably, we identified 100 significant proteins when comparing a high producer cell line with a low producer cell line, and 31 significant proteins when comparing a high producer cell line with a high producer cell line treated with tunicamycin (TM). Moreover, our research revealed distinct regulations of proteins associated with the ER stress response, mitochondrial unfolded protein response (UPRmt), and cell proliferation. Specifically, the cellular response to low dose tunicamycin treatment appeared to deviate from conventional expectations, indicating a potential pivotal role in translation processes before initiating the unfolded protein response (UPR) pathway. However, further investigations are required to fully elucidate this phenomenon. The application of label-free quantification (LFQ) allows the concurrent analysis of proteomics and phosphoproteomics profiles from identical samples without using additional chemical treatments and facilitating the interpretation of obtained proteomics results. This confers distinct analytical advantages. Moreover, LFQ is well-established, facilitating better comparability of generated data with those from other studies and research groups working on similar topics. The data presented in this paper underline the significance of LFQ proteomics analysis for obtaining a deeper understanding of diverse proteins and their responses to stress in CHO cells.

## Supplementary Information

Below is the link to the electronic supplementary material.

Supplementary file1 (XLSX 3108 KB)

## Data Availability

Proteomic data generated in this study have been deposited via ProteomeXchange, with identifier PXD049297.
